# Molecular targeting of the deubiquitinase USP14 to circumvent cisplatin resistance in ovarian carcinoma and identification of novel inhibitors

**DOI:** 10.20517/cdr.2025.88

**Published:** 2025-09-18

**Authors:** Cristina Corno, Debora Russo, Francesco Pignotti, Francesca De Giorgi, Ilaria Penna, Francesco Saccoliti, Matteo Costantino, Luca Mirra, Pietro Pettinari, Nives Carenini, Elisabetta Corna, Nunzio Perta, Chiara M Ciniselli, Pietro Pratesi, Rita Scarpelli, Fabio Bertozzi, Paolo Verderio, Giovanni L. Beretta, Giovanni Di Muccio, Daniele Di Marino, Tiziano Bandiera, Paola Perego

**Affiliations:** ^1^Unit of Molecular Pharmacology, Department of Experimental Oncology, Fondazione IRCCS Istituto Nazionale dei Tumori, Milan 20133, Italy.; ^2^D3-PharmaChemistry, Istituto Italiano di Tecnologia, Genoa 16163, Italy.; ^3^Department of Life and Environmental Sciences, Polytechnic University of Marche, Ancona 60131, Italy.; ^4^Medicinal Chemistry and Technologies for Drug Discovery and Delivery Facility, Istituto Italiano di Tecnologia, Genoa 16163, Italy.; ^5^Unit of Bioinformatics and Biostatistics, Department of Epidemiology and Data Science, Fondazione IRCCS Istituto Nazionale dei Tumori, Milan 20133, Italy.; ^6^(Present address) Structural Biophysics Facility, Istituto Italiano di Tecnologia, Genova 16163, Italy.; ^7^(Present address) Dipartimento di Scienze della Vita, della Salute e delle Professioni Sanitarie, Università degli studi Link Campus University, Roma 00165, Italy.; ^8^(Present address) Computational and Chemical Biology, Istituto Italiano di Tecnologia, Genova 16163, Italy.

**Keywords:** Deubiquitinases, cisplatin, drug resistance, proteasome

## Abstract

**Aim:** This study aims to investigate the biological role of the proteasome-associated deubiquitinase ubiquitin-specific protease 14 (USP14) in ovarian carcinoma drug resistance and to identify novel USP14 inhibitors (USP14i) for further preclinical development.

**Methods:** USP14 expression was evaluated in clinical samples from 134 ovarian carcinoma patients and in a broad panel of human ovarian carcinoma cell lines. Functional studies, including gain- and loss-of-function assays, migration and invasion, and apoptosis induction assays, were conducted using cisplatin-sensitive IGROV-1 cells and their cisplatin-resistant derivative IGROV-1/Pt1. A library of 1,056 small molecules was screened using an optimized hydrolysis assay. Docking and molecular dynamics simulations were employed to predict binding modes of candidate inhibitors within the USP14 domain.

**Results:** In clinical specimens, USP14 mRNA expression was associated with tumor grade. Exogenous overexpression of USP14 enhanced the survival of cisplatin-resistant IGROV-1/Pt1 cells, but not parental IGROV-1 cells, upon cisplatin exposure. USP14 knockdown by small interfering RNAs in resistant cells reduced aggressive features and restored cisplatin sensitivity, whereas no sensitization was observed in IGROV-1 cells. Medium-throughput screening identified five candidate molecules, among which ARN12502 showed the strongest inhibitory activity against USP14. ARN12502 exhibited an IC_50_ of 18.4 µM, and molecular dynamics simulations confirmed stable binding in two distinct modes. In proteasome sensor-expressing cells, ARN12502 displayed proteasome-inhibitory activity.

**Conclusion:** USP14 contributes to the aggressiveness of ovarian carcinoma, particularly to the cisplatin-resistant phenotype, and represents a relevant promising druggable target. ARN12502 serves as a starting point for chemical optimization toward the development of more potent USP14i.

## INTRODUCTION

Deubiquitinases (DUBs) are enzymes that remove ubiquitin (Ub) from target proteins^[1]^. This large protein family includes catalytically inactive members as well as three DUBs associated with the proteasome^[2]^. Evidence suggests that DUBs may be relevant therapeutic targets in cancer and other diseases, as their deregulation has been widely reported^[3,4]^. Altered DUB expression has been reported in both tumors and neurological disorders^[5,6]^. Moreover, DUBs are emerging as contributors to drug resistance in cancer^[5]^. In cancer, DUB inhibition is considered an opportunity to indirectly target proteins that are not easily druggable. For example, inhibition of ubiquitin-specific protease 7 (USP7), which regulates p53 levels, destabilizes MDM2 and thereby indirectly stabilizes p53^[7,8]^. Similarly, we recently showed that pharmacological or molecular inhibition of USP8 can reverse ovarian carcinoma cell resistance by deactivating HER family receptors^[9,10]^. Indeed, several DUBs are deregulated in ovarian carcinoma, where their expression has been associated with poor outcomes^[9,11]^.

Ovarian carcinoma is an aggressive disease with limited therapeutic options^[12]^. While first-line platinum-based therapy is effective, resistance frequently develops, highlighting the need to expand the therapeutic armamentarium^[12]^. Mechanisms underlying platinum resistance - including tumor-intrinsic factors and tumor microenvironment-related features - have been extensively characterized over the years, and new alterations continue to be identified^[13,14]^. DUBs are involved in multiple resistance mechanisms, as they regulate proteins critical for tumor survival during drug treatment, including mediators of apoptosis, DNA damage response and repair, and growth factor receptor-mediated signaling^[11]^.

The proteasome is a validated drug target in cancer, exemplified by the FDA approval of bortezomib (Velcade) for multiple myeloma in 2003^[15]^. Beyond direct proteasome inhibition, an alternative strategy is to target proteasome-associated DUBs within the 19S regulatory particle, namely USP14 and UCHL5^[16]^. The 19S particle contains three DUBs: the cysteine proteases UCHL5 and USP14, and the metalloprotease RPN11. USP14, in particular, has been reported to promote aggressive tumor behavior through multiple mechanisms. For instance, in hepatocellular carcinoma, USP14 enhances cancer cell growth by deubiquitinating HK2, AKT, and p62, thereby regulating glycolysis and autophagy^[17]^. In oral squamous cell carcinoma, USP14 deubiquitinates SOX2, promoting stem-like properties and tumor aggressiveness^[18]^.

Human USP14 is a monomeric protein of 494 amino acids, comprising an N-terminal Ub-like (UBL) domain (residues 1-93) and a C-terminal USP domain (residues 94-494)^[19]^. The UBL domain mediates recognition and interaction with targets such as the proteasome, while the USP domain provides catalytic activity^[20]^. Its catalytic triad consists of Cys144, His435, and Asp451.

Although growing evidence indicates a key role of USP14 in diverse physiological and pathological conditions, the development of selective USP14 inhibitors (USP14i) remains a challenging task, partly due to the high degree of conservation among DUBs. Early covalent and poorly selective inhibitors, such as b-AP15 and VLX-1570, showed promise but also significant limitations^[21,22]^. VLX-1570, for example, entered clinical trials for multiple myeloma but was discontinued due to toxicity^[23]^. Although a few USP14i have progressed into preclinical development, none have reached clinical testing. Therefore, there is an urgent need for novel, selective USP14i.

In this context, the present study was designed to investigate the biological role of USP14 in ovarian carcinoma and to identify new USP14i for evaluation in preclinical models.

## METHODS

### Clinical samples

Clinical samples from 134 consecutively recruited patients diagnosed with ovarian carcinoma were retrieved from the institutional biobank, which contains retrospectively collected samples. All patients provided informed consent for the use of their samples and clinical data in translational research. The study was approved by the review board of the Fondazione IRCCS Istituto Nazionale dei Tumori (protocol INT 23/21).

### Compounds and drugs

Compounds from the Istituto Italiano di Tecnologia (IIT, Genoa, Italy) library were dissolved in DMSO at a concentration of 10 mM and screened at a final concentration of 20 µM. IU1-47 (Sigma Aldrich, Milan, Italy) was dissolved in DMSO (10 mM) and diluted either in assay buffer for screening or in culture medium for cellular assays. Cisplatin (Teva Pharma Italia s.r.l., Milan, Italy) was diluted in saline.

### Cell lines and growth conditions

A broad panel of human ovarian carcinoma cell lines was used, including IGROV-1 cells and their cisplatin-resistant variant IGROV-1/Pt1, as previously described^[24]^; OVCAR-5, A2780, and their cisplatin-resistant variants (OVCAR-5/Pt, and A2780/CP)^[25]^; CaOv3, TOV112D, TOV21G, ES2, and SKOV3 (all from ATCC, Washington, DC, USA); PEO1 and PEO4 (Sigma/Aldrich); and OVCAR3, OVCAR4, and OVCAR8 (DCTD Tumor Repository, NCI, Frederick, MD, USA). Human osteosarcoma U2-OS cells were also purchased from ATCC. CaOv3 cells were cultured in Dulbecco’s Modified Eagle Medium (DMEM; Gibco, Carlsbad, California, USA) supplemented with 10% FBS (Gibco). PEO1 and PEO4 cells were maintained in RPMI-1640 medium (Gibco) supplemented with 10% FBS and 2 mM sodium pyruvate. ES2 and U2-OS cells were cultured in McCoy’s 5A medium (Gibco) with 10% FBS. All other cell lines were grown in RPMI-1640 medium supplemented with 10% FBS. All cultures were maintained for no more than 20 passages and were routinely checked for mycoplasma contamination (Lonza, Basel, Switzerland).

### Cell sensitivity assay

Colony-forming assays on plastic and soft agar were performed to evaluate cisplatin sensitivity. Cells were seeded in 6-well plates (100 cells/cm^2^) and, 24 h later, treated with cisplatin at different concentrations (IGROV-1: 2.5, 1.25, 0.625, 0.325, 0.1 µM; IGROV-1/Pt1: 10, 7.5, 5, 2.5, 1 µM). After 2 weeks of continuous exposure, colonies of ≥ 50 cells were fixed with alcohol, stained with 2% crystal violet (Sigma, Burlington, MA, USA), and counted. For soft agar assays, cells were exposed to cisplatin (100 µM) for 1 h, then seeded into 35-mm dishes (500 cells/well) in 0.33% agarose on a 0.5% agarose bed and incubated for ~2 weeks. Colonies were stained with p-iodonitrotetrazolium violet (Sigma) and counted 24 h later under a magnifying projector. IC_50_ values were defined as the drug concentrations that reduced cell survival by 50%.

### Quantitative real-time PCR

Total RNA was isolated using the RNeasy Plus Mini Kit (Qiagen, Hilden, Germany). One microgram of RNA was reverse transcribed in the presence of RNase inhibitors using the High-Capacity cDNA Reverse Transcription Kit (Thermo Fisher Scientific, Waltham, MA, USA), according to the manufacturer’s protocol. USP14 expression was quantified by relative quantification (RQ) using the comparative Ct (ΔΔCt) method, as described in the Supplementary Materials.

### Gain-of-function studies

USP14 overexpression was achieved by lentiviral infection of IGROV-1 and IGROV-1/Pt1 cells. Cells were seeded in 6-well plates and, once confluent, infected with lentiviral particles (USP14-Myc-DDK tagged or control; Origene, Tema Ricerca, Milan, Italy) for 48 h, following the manufacturer’s instructions. Selection with puromycin (5 μg/mL) was initiated 72 h after infection. Transgene expression was verified by Western blotting as indicated below.

### Loss-of-function studies

Two small interfering RNAs (siRNAs) targeting USP14 [Silencer Select s17358 (siRNAa) and s17360 (siRNAb); Thermo Fisher Scientific], along with a negative control siRNA (Silencer Select Negative Control #2 siRNA), were used. IGROV-1 and IGROV-1/Pt1 cells were seeded in 6-well plates and transfected 24 h later with 10 nM siRNAs in Opti-MEM medium (Gibco) using Lipofectamine RNAiMAX (Thermo Fisher Scientific). After 5 h, Opti-MEM was replaced with complete medium. USP14 levels were evaluated by quantitative real-time PCR (qRT-PCR) 48-72 h after transfection. At 48 h, cells were harvested for: (a) colony-forming assays in plastic and soft agar to assess cisplatin sensitivity; (b) migration and invasion assays in 24-well transwell chambers; and (c) Annexin V-binding assays in 6-well plates.

### Migration and invasion assays

Cells were seeded into 24-well transwell chambers (Corning, NY, USA) in serum-free medium 48 h after transfection. For invasion assays, transwells were precoated with 12.5 mg Matrigel/well (BD Biosciences, Franklin Lakes, NJ, USA) and allowed to dry for 1 h before seeding. After 24 h, cells were fixed with ethanol and stained with 0.4% sulforhodamine B (Sigma) in 0.1% acetic acid. Migrated or invading cells on the lower side of the membrane were counted (five random fields/sample) under an inverted microscope.

### Western blot analysis

Cells were lysed, and protein extracts were analyzed by western blotting analysis according to standard protocols (details in Supplementary Materials).

### Annexin V-binding assay

To quantitatively assess apoptosis after cisplatin exposure, Annexin V-binding assays (Immunostep, Salamanca, Spain) were performed. Forty-eight hours after transfection, cells were treated with cisplatin for 1 h. Twenty-four hours later, cells were washed with PBS, processed according to the manufacturer’s protocol, and examined by flow cytometry (BD Accuri C6, Becton, Dickinson, Franklin Lakes, NJ, USA). Data analysis was carried out using the instrument software on a minimum of 10,000 acquired cells per sample.

### Medium-throughput screening for USP14i

A medium-throughput screen of 1,056 compounds from the IIT compound collection was conducted to identify small molecules capable of inhibiting proteasome-associated USP14. Screening employed an optimized hydrolysis assay originally based on Ub-AMC^[26]^, which was adapted to a 384-well format (384 Well Microplate, PS, Small Volume™, HiBase, Non-Binding, 784900, Greiner) using a commercial recombinant enzyme with the fluorogenic substrate Ub-Rho110-G. In a 15 µL reaction volume, 60 nM USP14 [USP14 (6His-tagged), 64-0018-050, Ubiquigent, Dundee, UK] was preincubated in assay buffer (50 mM Hepes pH 7.5, 1 mM EDTA, 0.1% BSA, 1 mM DTT, 1 mM ATP-MgCl_2_ and 0.01% Tween-20) with 0.75 nM ubiquitin vinyl sulfone-treated human proteasome [Ub-VS 26S, 26S Proteasome (Ub-VS-treated), 65-1020-010, Ubiquigent] for 15 min at 25 °C. Then, test compounds were added at a final concentration of 20 µM in technical duplicates and incubated with the enzyme/proteasome mix for 30 min at 25 °C. The reaction was initiated by adding Ub-Rho110-G at a final concentration of 1 μM, and substrate cleavage was measured after 35 min at 25 °C by detecting fluorescence (excitation 485 nm, emission 535 nm) using an EnVision 2104 plate reader (PerkinElmer, Waltham, USA) equipped with an appropriate dichroic mirror (e.g., FITC D505).

Compound dilutions for single-concentration screening were prepared using a Hamilton Robotics Microlab STAR Plus workstation (8- and 96-channel Co-RE head dispensing, IS-WAP Gripper, and CO-RE Gripper robotic arms). Screening plates (384-well) were prepared using a three-step custom automated method: (1) dilution of compounds in DMSO; (2) further dilution in assay buffer; and (3) transfer of diluted compounds from 96- to 384-well plates.

Each screening plate included controls in the first and last columns. Negative controls (0% inhibition) consisted of reactions without compound (DMSO only, in the presence of USP14 + Ub-VS 26S, named as Total Activity), USP14 or Ub-VS 26S alone, and blanks (assay buffer only). The USP14 reference inhibitor IU1-47 was used as a positive control at final concentrations of 200 nM and 10 µM, producing 50% and 100% inhibition, respectively.

Hits were defined as compounds producing over 20% of USP14 inhibition. Initial hits were retested at five concentrations (1, 10, 20, 30, and 55 µM) to confirm activity. ARN12502 emerged as the most potent compound. To measure the IC_50_ of ARN12502 (the concentration causing half-maximal inhibition), dose-response curves were generated by non-linear regression analysis of Log[concentration] versus response, using mean replicate values fitted to a four-parameter Hill equation (GraphPad Prism 8, GraphPad Software Inc., CA, USA).

### USP14 activity and proteasome perturbation

USP14 activity, under basal conditions or after treatment with inhibitors, was assessed as described in Corno *et al.*^[9]^. An antibody against USP14 (Bethyl Laboratories) was used to detect both labeled and unlabeled USP14 by Western blotting.

Proteasome perturbation was evaluated using the U2-OS cell line stably transfected to express Zoanthus sp. Green Fluorescent Protein (ZsGreen) fused to the mouse ornithine decarboxylase degradation domain (pZsProSensor-1, Takara Bio, Orchard Parkway, San Jose, CA, USA). U2-OS polyclones (U2-OS/pZS) were selected in the presence of 400 µg/mL G418. Cells were exposed for 24 h to IU1-47 and ARN12502, and proteasome perturbation was confirmed by increased fluorescence.

### Docking and molecular dynamics simulations

For docking and simulation procedures, the USP domain of USP14 from the cryo-EM structure PDB:7W3A was used^[19]^. The binding pocket was identified by blind docking using two different software programs, Glide and DiffDock^[27,28]^, supported by literature evidence^[29,30]^. Initial configurations for molecular dynamics (MD) simulations were prepared by targeted docking with Glide around the identified pocket. Simulations were performed using the GROMACS version 2023.2^[31]^, and ligand-protein contact analyses were carried out with the ProLif Python package^[32]^. The AMBER-ff19SB force field^[33]^ with the TIP3P water model^[34]^ was applied. Parameterization of ARN12502 was conducted using Gaussian 09^[35]^ for QM calculations at the HF/6-31G* level, followed by RESP charge computation using Antechamber^[36]^. Internal parameters were assigned using tleap with the GAFF2 force field^[37,38]^. Simulation boxes were generated using the solution builder tool on the CHARMM-GUI website^[39]^, using KCl to neutralize the total charge and set the ionic concentration to 0.150 M. After energy minimization (steepest descent), a multistep equilibration protocol adapted from reference^[40]^ was applied for 20 ns, consisting of alternating NPT and NVT simulations. Production runs were carried out in three replicas of 1 µs each (total 3 µs) at 300 K and 1 bar. The most representative conformation was identified by RMSD clustering of the trajectory using the GROMACS cluster tool with the Gromos method and a cutoff of 0.3 nm. Visual inspection of simulations was performed with VMD software^[41]^, and image rendering was done using ChimeraX^[42]^.

### Statistical analysis of preclinical data and human patients

Associations in preclinical experiments were evaluated with the Wilcoxon (W) or Kruskal-Wallis (KW) non-parametric test, depending on the number of groups. Exact (E) or Monte Carlo (MC) approaches were used to estimate the corresponding p-values. Corrections for multiple comparisons were performed using the Bonferroni adjustment. For the evaluation of USP14 expression in the study cohort of 134 ovarian carcinoma patients, medians and ranges were used to summarize continuous variables, and frequency tables were used for categorical variables. Statistical analysis was based on the log_2_RQ distribution of USP14 relative to the mean expression values of two housekeeping genes (GAPDH and RSP13), calculated as:

**Figure:**
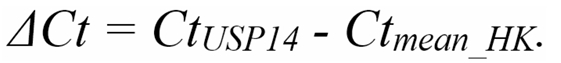


Associations between the main clinicopathological characteristics and USP14 levels were tested with the KW- or W- tests. Univariate logistic regression models were also applied, with results reported as odds ratio (OR) and 95% confidence interval (95%CI). All statistical analyses were performed with SAS software (SAS^®^ Studio, Basic, version 5.2, SAS Institute Inc., Cary, NC, USA), adopting a nominal α level of 5%. Boxplots were generated using the R software (R Foundation for Statistical Computing, Vienna, Austria) with the ggplot2 package.

## RESULTS

### Expression of USP14 in a patient cohort and in various ovarian carcinoma cell lines

**Figure 1:**
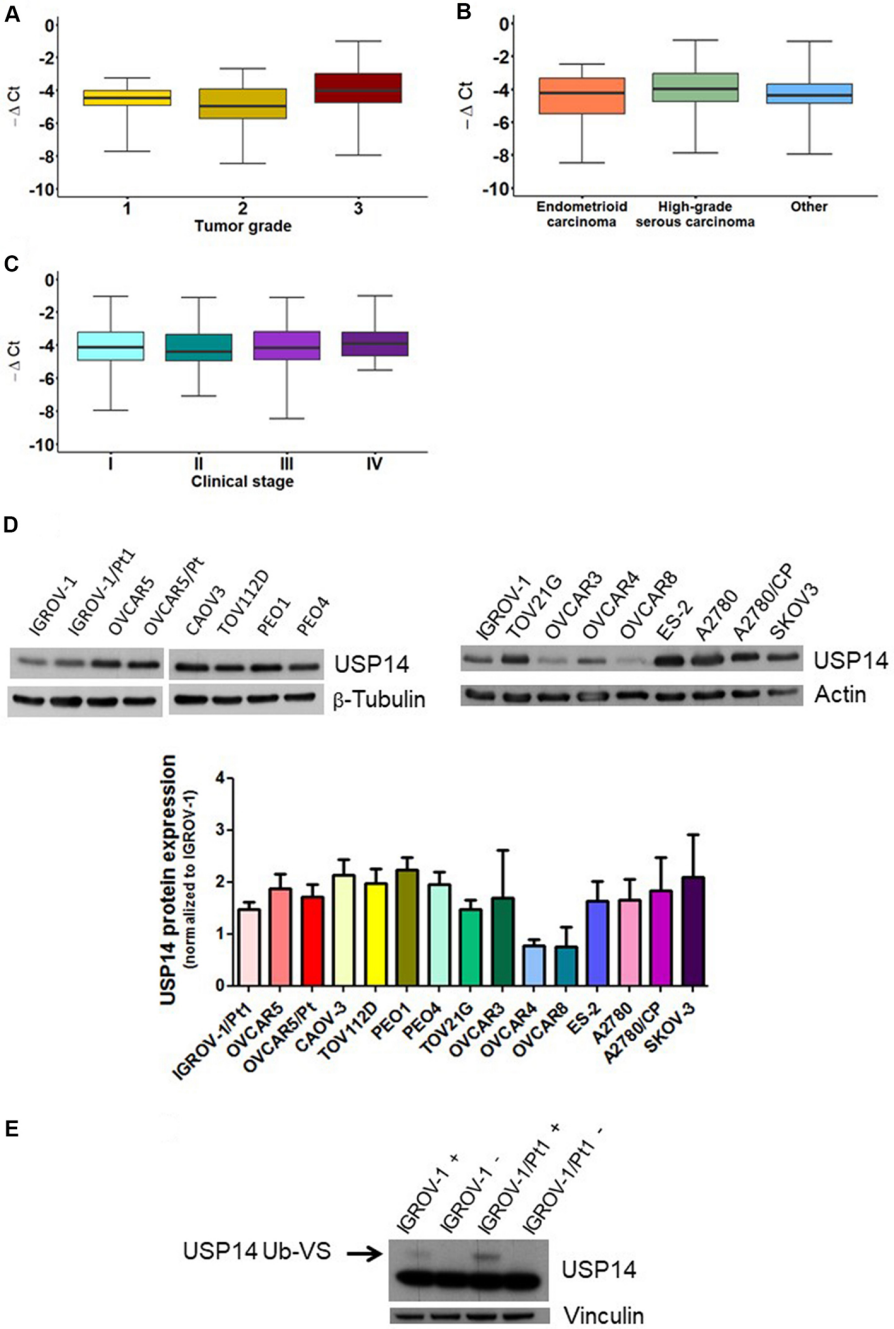
Relative expression of USP14 in ovarian carcinoma patients and USP14 protein expression and activity in different ovarian carcinoma cell lines. Box plots show USP14 expression according to (A) tumor grade, (B) diagnosis, and (C) stage. Each box represents the 25th-75th percentile range, with the horizontal line indicating the median and whiskers showing the extreme values; (D) Western blot analysis of USP14 protein expression in various ovarian carcinoma cell lines and their resistant variants. Actin or β-tubulin served as loading controls. Band intensities were quantified using ImageJ, normalized to the loading control, and expressed relative to IGROV-1. The histogram below reports mean ± standard deviation (SD) from three technical replicates; (E) USP14 activity in IGROV-1 and IGROV-1/Pt1 cells. Deubiquitinase labeling with HA-Ub-VS was followed by SDS-PAGE and immunoblotting with USP14. +, samples incubated with HA-Ub-VS; -, samples incubated without HA-Ub-VS. Vinculin served as loading control; the active band is indicated by the arrow. USP14: Ubiquitin-specific protease 14; HA-Ub-VS: hemagglutinin-ubiquitin-vinyl sulfone.

To gain insights into the role of USP14 in ovarian carcinoma and assess its potential as a therapeutic target, we first examined its expression in clinical specimens. A cohort of 134 ovarian carcinoma patients was analyzed, and the main clinicopathological characteristics are reported in Supplementary Table 1. Most patients had high-grade serous ovarian carcinoma (60%), Grade 3 tumors (70%), and Stage III disease (53%). An association between tumor grade and USP14 levels was observed (Figure 1A, KW *P* = 0.018). This association persisted when data were dichotomized (Grade 1+2 *vs.* Grade 3; Supplementary Figure 1) and included in a univariate logistic regression model (OR: 0.621, 95%CI: 0.452-0.854). By contrast, no association was found between USP14 levels and diagnosis (Figure 1B, KW *P* = 0.233) or stage (Figure 1C, KW *P* = 0.794).

We next evaluated the expression and activity of USP14 in different ovarian carcinoma cell lines, including cisplatin-resistant variants. USP14 mRNA levels varied across the cell line panel [Supplementary Figure 2]. Western blot analyses indicated that all ovarian carcinoma cell lines expressed USP14, with protein levels differing among them [Figure 1D]. Notably, the cisplatin-resistant subline IGROV-1/Pt1 displayed higher USP14 protein levels compared with the cisplatin-sensitive parental line IGROV-1 [Figure 1D]. We further assessed USP14 enzymatic activity in this sensitive-resistant pair using the HA-Ub-VS probe, which targets the active site of DUB enzymes. USP14 activity was increased in IGROV-1/Pt1 cells compared with IGROV-1 cells [Figure 1E]. In other resistant sublines, USP14 protein levels did not differ significantly from those of their respective parental cells.

### Overexpression of USP14 in sensitive and resistant cell lines

**Figure 2:**
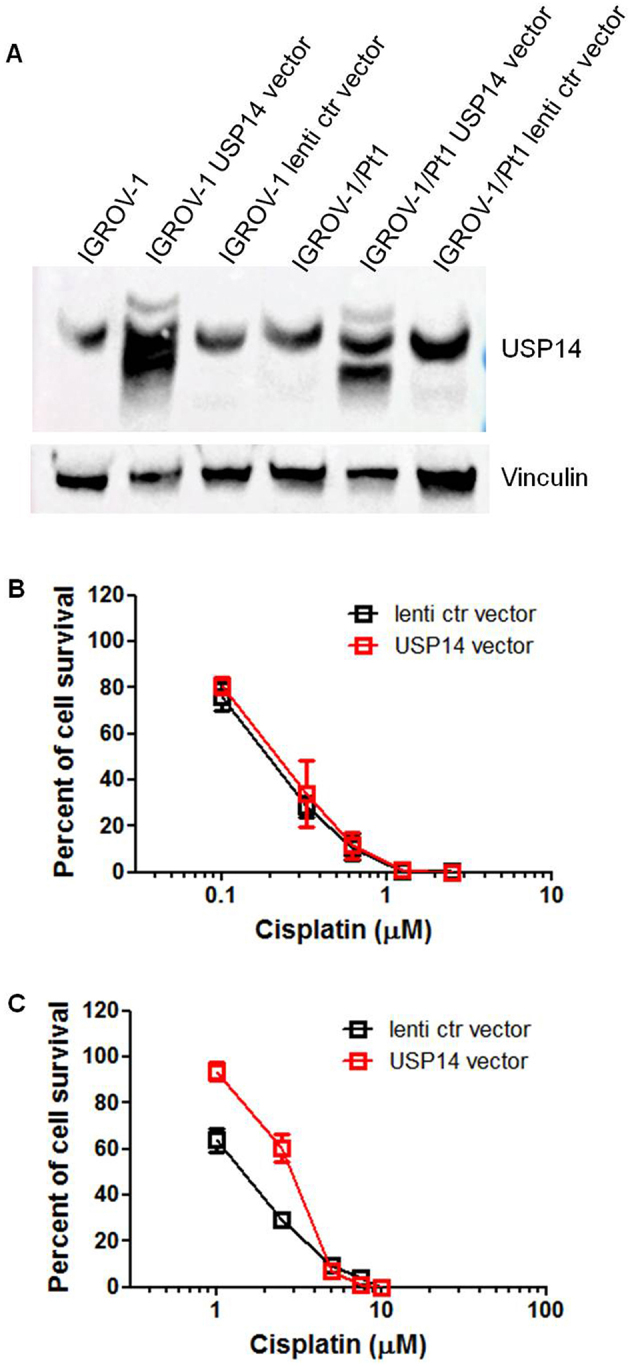
Colony-forming ability of IGROV-1 and IGROV-1/Pt1 cells overexpressing USP14. (A) Western blot analysis of USP14 protein expression in IGROV-1 and IGROV-1/Pt1 cells stably transfected with lentiviral particles carrying USP14 or a lentiviral control (ctr) vector; (B) IGROV-1 and (C) IGROV-1/Pt1 cells were seeded in 6-well plates for colony-forming assays on plastic. Twenty-four hours after seeding, cells were treated with cisplatin and incubated for 10 days. IC_50_ value is the concentration inhibiting cell growth by 50% (IGROV-1: lenti ctr vector, IC_50_ = 0.2 ± 0.01 µM; USP14 vector, IC_50_ = 0.203 ± 0.087 µM; IGROV-1/Pt1: lenti ctr vector, IC_50_ = 1.43 ± 0.245 µM; USP14 vector, IC_50_ = 2.79 ± 0.29 µM; one-sided KW_E_ *P* = 0.05). USP14: Ubiquitin-specific protease 14.

Given that USP14 levels were increased in the cisplatin-resistant IGROV-1/Pt1 variant compared to the parental IGROV-1 cells, this cell pair was selected for gain-of-function studies. Cells were transduced with lentiviral particles containing USP14 cDNA or a control vector. In the obtained cells [Figure 2A, Supplementary Figure 3], cisplatin sensitivity was assessed using a colony-forming assay on plastic. IC_50_ values were comparable between parental IGROV-1 lentiviral control cells and those overexpressing exogenous USP14 (0.203 ± 0.087 *vs.* 0.2 ± 0.01 µM, Figures 2B and C). In contrast, IGROV-1/Pt1 cells overexpressing USP14 showed increased resistance, with an IC_50_ of 2.79 ± 0.29 µM compared to 1.43 ± 0.245 µM in control cells (one-sided KW_E_ *P* = 0.05).

### Effect of USP14 silencing on resistance and aggressive phenotype of IGROV-1/Pt1 cells

**Figure 3:**
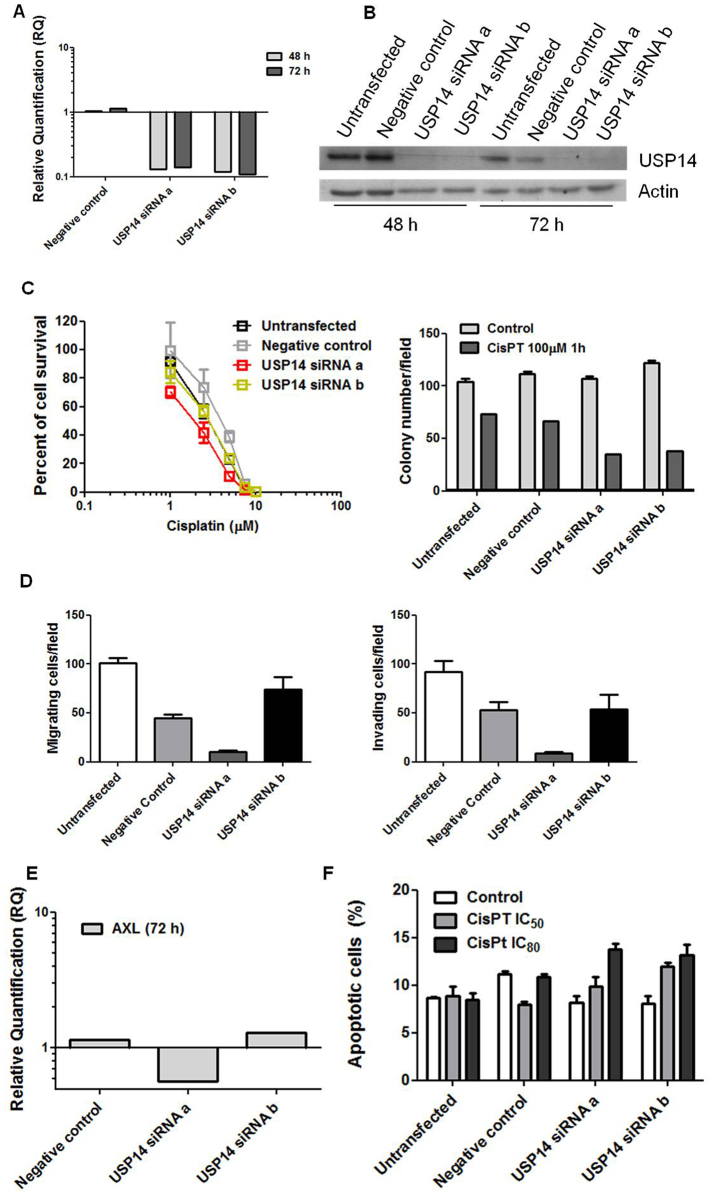
Molecular targeting of USP14 in IGROV-1/Pt1 cells. (A) Quantitative RT-PCR analysis of USP14 mRNA levels after siRNA transfection; untransfected cells were used as calibrator, with GAPDH as housekeeping gene; (B) Western blot analysis of USP14 protein levels at different time points after siRNA transfection, with actin as loading control; (C) Colony-forming ability on plastic (left) and in soft agar (right) 48 h after transfection. For the plastic clonogenic assay, cells were continuously exposed to cisplatin and counted after 10 days. IC_50_ is the concentration inhibiting cell growth by 50%: untransfected, 2.9 µM; negative control, 4.1 µM; USP14 siRNAa, 1.95 µM; USP14 siRNAb, 2.9 µM. For the agar assay, cells were treated with 100 µM cisplatin for 1 h, seeded in 0.33% agarose on a 0.5% agarose bed, and incubated for 2 weeks. Cell survival rates (treated versus control): 70% (untransfected), 60% (negative control), 33% (USP14 siRNAa), and 31% (USP14 siRNAb). Histograms show mean ± SD of at least three technical replicates; (D) Migratory and invasive ability of USP14-silenced cells 48 h after transfection using transwell chambers in serum-free medium. After 24 h, cells were fixed in ethanol, stained with 0.4% SRB, and counted under an inverted microscope. Graphs report mean ± SD of at least three technical replicates; error bars are not visible when values are nearly identical; (E) Quantitative RT-PCR of Axl mRNA levels after siRNA transfection. Untransfected cells were used as calibrator, and GAPDH served as housekeeping gene; (F) Apoptosis analysis in USP14-silenced cells. IGROV-1/Pt1 cells were seeded 48 h after transfection start and treated with cisplatin at IC_50_ and IC_80_ doses for 1 h, and apoptosis was assessed 24 h later by Annexin-V binding assay. Columns indicate total apoptosis. USP14: Ubiquitin-specific protease 14; RT-PCR: real-time PCR; siRNA: small interfering RNA; siRNAa: Silencer Select s17358; siRNAb: Silencer Select s17360; SD: standard deviation.

We next examined the effects of USP14 knockdown in IGROV-1/Pt1 cells to clarify its contribution to drug resistance and aggressive features. Transient transfection with two distinct siRNAs (a and b) targeting USP14 markedly reduced USP14 mRNA and protein levels at different time points [Figure 3A and B]. USP14 silencing increased cisplatin sensitivity, as shown by clonogenic assays on plastic [Figure 3C]. Enhanced cisplatin susceptibility upon USP14 knockdown was further confirmed in soft agar assays (KW_MC_ *P* < 0.001), with significant effects observed for both siRNAs compared with negative controls (one-sided contrast W_MC_ *P* < 0.001, Figure 3C). As shown previously, IGROV-1/Pt1 cells exhibit significantly higher migratory and invasive properties compared to parental IGROV-1 cells^[43]^. To investigate the impact of USP14 knockdown on these aggressive features, we performed transwell assays. USP14 knockdown significantly affected migration and invasion (KW_MC_ *P* < 0.001, Figure 3D). Specifically, siRNAa reduced the ability to migrate and invade compared with negative control cells (one-sided contrast, Bonferroni W_E_ *P* < 0.001) and significantly decreased aggressiveness compared to untransfected cells (one-sided contrast, Bonferroni W_E_ *P* < 0.001). A similar trend was observed with siRNAb, although statistical significance was not reached after Bonferroni correction (one-sided contrast, Bonferroni-adjusted W_E_ *P* = 0.129, Figure 3D). These changes were associated with decreased Axl expression [Figure 3E]. Consistently, cisplatin-induced apoptosis was increased in USP14-silenced cells (KW_E_ *P* = 0.074, 0.026, and < 0.001 for control, cisplatin IC_50_, and cisplatin IC_80_, respectively, Figure 3F).

To further explore the pathways affected, an antibody array was used to examine apoptosis-related factors following USP14 knockdown in IGROV-1/Pt1 cells. As shown in Supplementary Figure 4, unlike cisplatin-treated negative control siRNA-transfected cells, USP14-silenced siRNA-transfected IGROV-1/Pt1 cells exposed to cisplatin displayed decreased levels of the antiapoptotic proteins Bcl-x_L_ and survivin, consistent with enhanced drug-induced apoptosis.

### Molecular inhibition of USP14 in cisplatin-sensitive IGROV-1 cells

**Figure 4:**
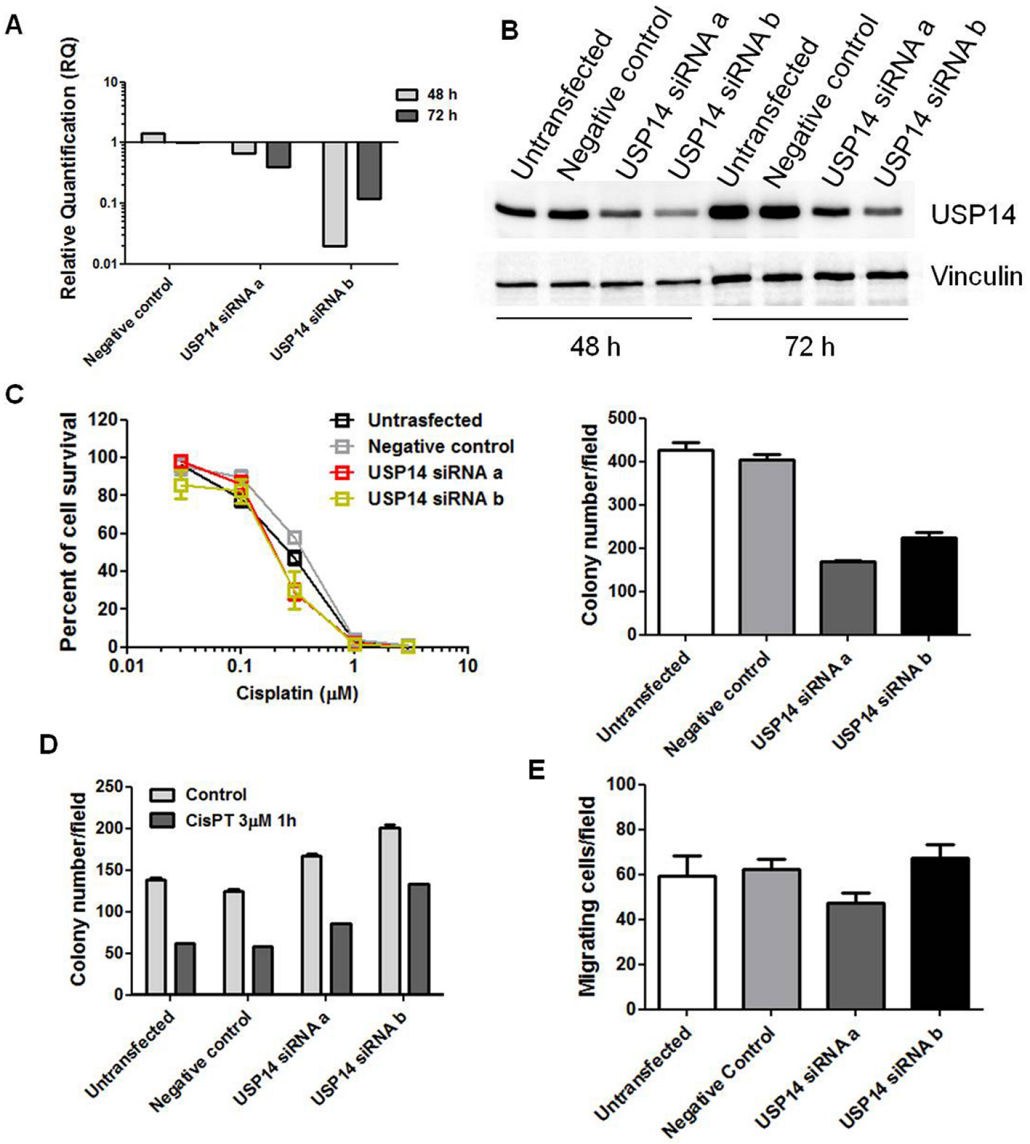
Molecular targeting of USP14 in IGROV-1 cells. (A) Quantitative RT-PCR analysis of USP14 mRNA levels after siRNA transfection; untransfected cells were used as calibrator, with GAPDH as housekeeping gene; (B) Western blot analysis of USP14 protein levels at different time points after siRNA transfection, with actin as loading control; (C and D) Colony-forming ability on plastic; (C) and in soft agar (D) 48 h after transfection. Graphs report mean ± SD of at least three technical replicates. For the plastic assay, cells were continuously exposed to cisplatin and counted after 10 days. IC_50_ is the concentration inhibiting cell growth by 50%: untransfected, 0.27 µM; negative control, 0.36 µM; USP14 siRNAa, 0.20 µM; USP14 siRNAb, 0.19 µM. The histogram (right) shows colony numbers of untreated cells (mean ± SD, ≥ 3 technical replicates). For the agar assay, cells were treated with 3 µM cisplatin for 1 h, then seeded in 0.33% agarose on a 0.5% agarose bed, and incubated for 2 weeks. Cell survival (treated versus control): 45% (untransfected), 46% (negative control), 51% (USP14 siRNAa), and 66% (USP14 siRNAb); (E) Migratory ability of USP14-silenced cells assessed 48 h after transfection using transwell chambers in serum-free medium. After 24 h, cells were fixed in ethanol, stained with 0.4% SRB, and counted under an inverted microscope. Columns show mean ± SD of three technical replicates. USP14: Ubiquitin-specific protease 14; siRNA: small interfering RNA; siRNAa: Silencer Select s17358; siRNAb: Silencer Select s17360; SD: standard deviation.

The effects of USP14 inhibition were also assessed in cisplatin-sensitive IGROV-1 cells. Both siRNAs effectively suppressed DUB expression [Figure 4A and B] compared with untransfected and negative control siRNA-transfected cells, as demonstrated by qRT-PCR and western blot analysis. USP14 silencing markedly reduced colony-forming ability on plastic (KW_E_ *P* < 0.001, Figure 4C). However, cisplatin sensitivity was not significantly altered [Figure 4C and D], as shown by both plastic and agar assays. We further examined cell migration following USP14 knockdown in IGROV-1 cells but observed no significant differences (KW_MC_ *P* = 0.07, Figure 4E).

### Identification of ARN12502

Measuring the specific activity of USP14 toward fluorogenic substrates (e.g., Ub-Rho110-G) is challenging, as free USP14 exhibits minimal DUB activity in the absence of the proteasome. Moreover, UCH37, another proteasome-associated DUB, displays higher activity than USP14 on fluorogenic Ub adducts. To overcome these limitations and specifically assess USP14 activity, we employed a “Poisoned Proteasome” strategy. Treatment of the 26S proteasome with Ub-VS irreversibly inhibits both UCH37 and USP14 by forming a covalent adduct with the active-site cysteine in thiol protease-class DUBs. Reconstitution of Ub-VS-treated 26S with recombinant USP14 subsequently results in a ~800-1,000-fold increase in USP14 DUB activity compared with free USP14^[44]^. Using this optimized Ub-AMC hydrolysis assay, we performed a medium-throughput screen to identify novel inhibitors of proteasome-associated USP14. A small subset of the IIT chemical library, which comprises > 13,000 natural and synthetic compounds, was tested.

**Figure 5:**
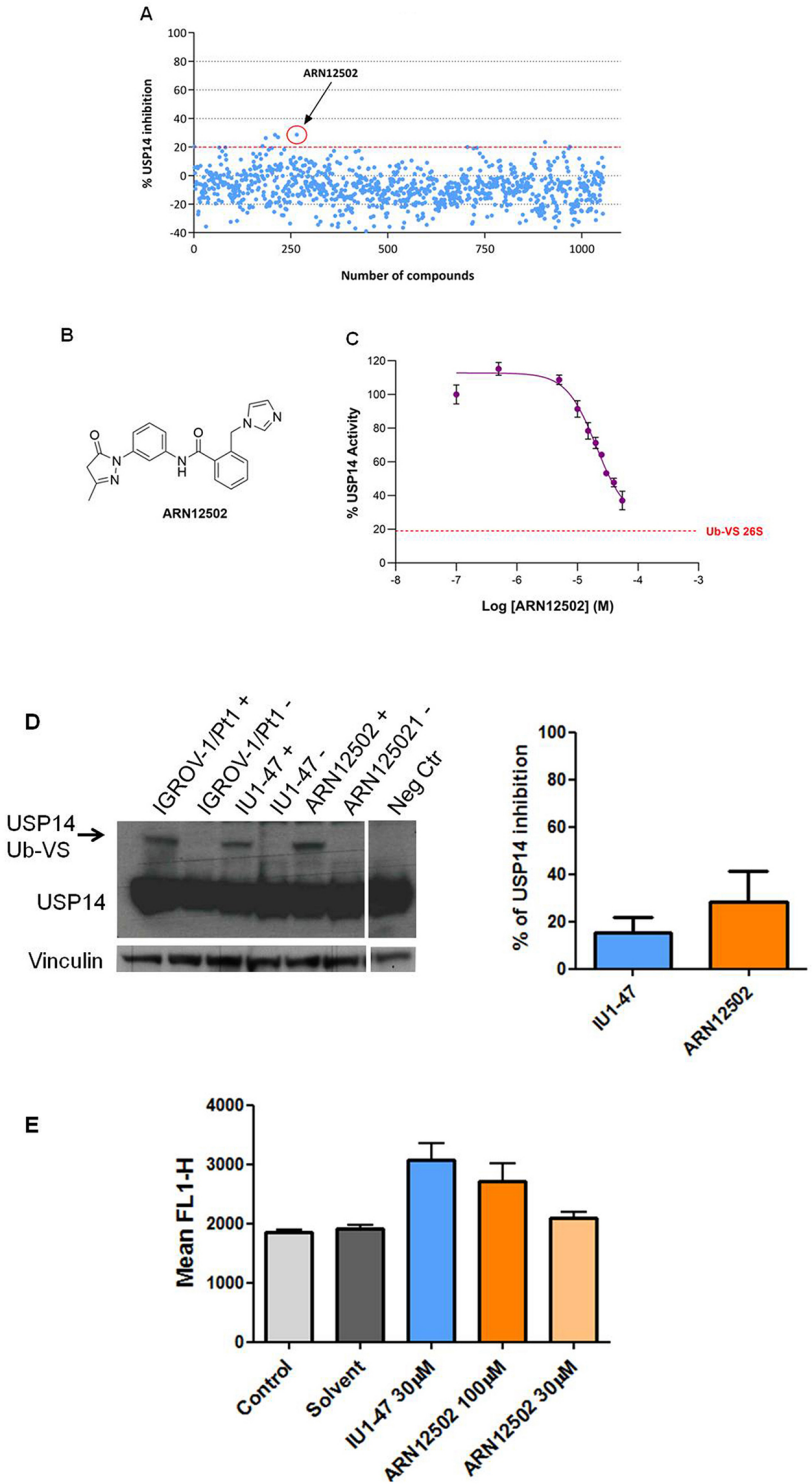
Identification of new USP14i. Medium-throughput screening for USP14i was performed using Ub-Rho110-G hydrolysis assay. (A) Primary screening results of 1,056 compounds at 20 µM; (B) Chemical structure of ARN12502; (C) Representative inhibition curve of ARN12502. The dotted red line indicates basal Ub-VS 26S activity (~20% of total measured activity). The dose-response curve is representative of three independent experiments; (D) USP14 activity in IGROV-1/Pt1 cells after treatment with USP14i. Deubiquitinases were labeled with HA-Ub-VS, followed by western blotting. Cells were treated with 100 µM IU1-47 or ARN12502 for 24 h. +, samples incubated with HA-Ub-VS; -, samples incubated without HA-Ub-VS. Vinculin served as loading control. The histogram reports percentage inhibition of USP14 activity (mean ± SD, three technical replicates); (E) Proteasome perturbation analysis in U2OS/pZS cells using flow cytometry after treatment with USP14i for 24 h. IU1-47 was used as reference. Values represent mean ± SD of three technical replicates. USP14: Ubiquitin-specific protease 14; USP14i: USP14 inhibitors; SD: standard deviation; HA-Ub-VS: hemagglutinin-ubiquitin-vinyl sulfone.

The initial screening subset of 1,000 small molecules was generated by applying hierarchical agglomerative cluster analysis to ~13,300 compounds, as previously described^[45]^. The agglomerative process was arbitrarily terminated when 1,000 clusters had been formed, and the centroid compound of each cluster was selected as its representative. Visual inspection of these compounds guided the selection of a second, smaller subset. A number of compounds were filtered out due to structural similarity or complex chemical structures. Additional candidates were selected from the IIT library based on their similarity to known DUB inhibitors reported in the scientific and patent literature, such as IU1 and IU1-47, and added to the second set. In total, 1,056 compounds were assembled for testing [Figure 5A]. For the screening, 176 compounds per plate were assayed in duplicate at a single concentration (20 µM). Using a threshold of ~20% inhibition, five hits were identified and subsequently confirmed by retesting at five concentrations (1, 10, 20, 30, and 55 µM). The most active compound, ARN12502 [Figure 5B], was then tested in a dose-response experiment over the range 0.5-55 µM, yielding an IC_50_ of 18.4 ± 2.2 µM. A representative inhibition curve is shown in Figure 5C.

We next assessed USP14 activity in IGROV-1/Pt1 cells treated with ARN12502 for 24 h and observed a modest reduction in active USP14 compared with the reference compound IU1-47 [Figure 5D]. Given that USP14 is a proteasome-associated DUB, we analyzed whether USP14 inhibition resulted in proteasome perturbation in the U2OS-pZS cell line using a proteasome sensor vector assay (KW_E_ *P*-value: 0.01, Figure 5E). At the highest concentration, ARN12502 enhanced fluorescence relative to controls, although to a lesser extent than IU1-47, indicating partial proteasome perturbation.

### Computational analysis

**Figure 6:**
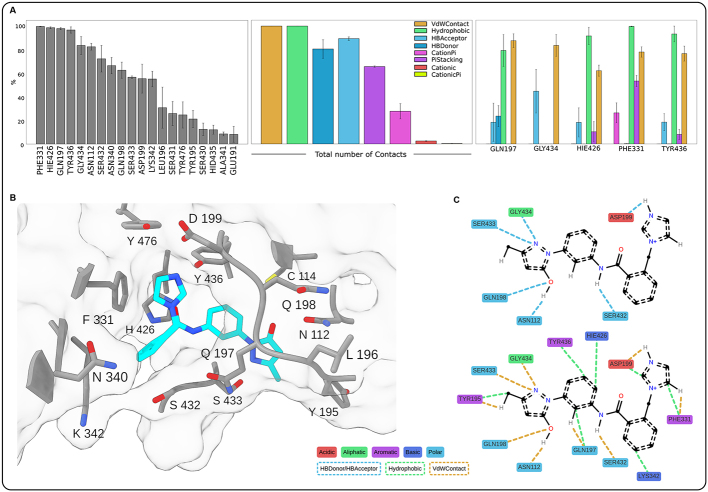
Contact analysis of the ARN12502-USP14 complex from molecular dynamics (MD) simulations. (A) Bar plots showing interactions of ARN12502 with USP14, averaged over three replicates; error bars represent standard error. The left panel shows the persistence of contacts (% simulation time) between the ligand and the top 20 residues. The middle and right panels report the percentage of trajectory frames in which each interaction type was present, globally and by residue (top five mostly contacted), respectively; (B) 3D illustration of the most representative structure obtained by RMSD clustering, with ARN12502 in cyan and surrounding USP14 residues in grey, showing the coordination of the ligand inside the binding pocket; hydrogen atoms are omitted for clarity; (C) Schematic 2D representation corresponding to panel B, showing specific contacts; only interacting hydrogens of ARN12502 are displayed. Interaction types and residue classes are indicated in the legend. For clarity, representation is split into two panels. USP14: Ubiquitin-specific protease 14; 3D: three-dimensional; 2D: two-dimensional.

To investigate the molecular mechanism of USP14 inhibition by ARN12502, we carried out computational analyses including docking and molecular dynamics simulations. The docking procedure revealed that ARN12502 binds to the same pocket of USP14 that is targeted by known inhibitors of the IU family [Supplementary Figure 5]. This pocket is formed by the blocking loop 1 (BL1) and blocking loop 2 (BL2) of the USP domain, and is located near the catalytic triad of USP14 (Cys114, His435, Asp451) [Supplementary Figure 6]. The predicted docking complex indicated that ARN12502 interacts with several residues also contacted by IU1, as His426 and Tyr436^[30]^. Due to the dynamic nature of BL1 and BL2, as well as the conformational flexibility of ARN12502, we performed two sets of molecular dynamics simulations, starting from two distinct docking poses with different affinity scores [Supplementary Figure 7]. As expected, in the low-affinity pose, ARN12502 exited the binding site before 400 ns of simulation. By contrast, in the high-affinity pose, ARN12502 remained stably bound within the pocket, displaying a very low RMSD (0.28 ± 0.05 nm) and a short ligand-pocket distance (0.50 ± 0.04 nm) [Supplementary Figure 8]. In this stable binding mode, ARN12502 maintained persistent interactions with residues Phe331, His426, Gln197, and Tyr426 throughout nearly the entire simulation [Figure 6A]. It also engaged with Gly434, Asn112, and Ser432 for more than 70% of the time. A more detailed analysis [Figure 6A] revealed that these residues primarily established hydrophobic and van der Waals (vdW) contacts, with occasional hydrogen bonds (Tyr436, His426, Gly434, Gln197), π-stacking interactions (Phe331, His426, Tyr426), and cation-π interactions (Phe331). Overall, vdW and hydrophobic interactions were consistently present during the simulation, while H-bonds and π-stacking interactions occurred slightly less frequently, with occupancies between 55% and 90%. The time-dependent profiles of these contacts are presented in Supplementary Figure 8. Analysis of a representative binding pose [Figure 6B and C] highlights the spatial arrangement of key residues mediating ARN12502 binding. Notably, hydrogen bonds were observed with Ser432, Asn112, Gln198, Ser433, Asp199, and Ser434.

## DISCUSSION

DUBs have been regarded as promising therapeutic targets in cancer due to their dysregulation. Although early evidence supports a role for several DUBs in specific cancer types, the development of DUB inhibitors remains in its infancy, with only a limited number reaching clinical evaluation. Recent trials have shown mixed outcomes - for example, the USP1 inhibitor TNG348 was terminated for safety reasons (ClinicalTrials.gov; NCT06065059), while other selective inhibitors are still under evaluation.

In this study, we investigated the role of USP14 in ovarian carcinoma, beginning with the analysis of clinical specimens. We found that USP14 mRNA levels correlated with tumor grade, consistent with previous reports linking high USP14 expression to poor prognosis in ovarian carcinoma patients^[46]^. Moreover, analysis of the TCGA database revealed an association between elevated USP14 mRNA levels and shorter progression-free survival (data not shown). To further explore the contribution of USP14 to ovarian cancer aggressiveness, we profiled several ovarian carcinoma cell lines. We observed enhanced USP14 protein levels in the cisplatin-resistant IGROV-1/Pt1 cell model compared to the parental IGROV-1 cells. This model is considered clinically relevant as it harbors *TP53* mutations and exhibits hyperactivation of the canonical MAPK pathway^[24,25]^.

Knockdown of USP14 in cisplatin-resistant IGROV-1/Pt1 cells enhanced drug-induced apoptosis, indicating an interplay between USP14 and components of the apoptotic pathway in resistant cells, since this effect was not observed in cisplatin-sensitive IGROV-1 cells (data not shown).

The distinct effects of USP14 silencing or overexpression in parental versus resistant cells might be dependent on the different molecular backgrounds, implying that USP14 function is regulated by interactions with specific protein partners. Supporting this view, enhanced apoptosis upon USP14 knockdown by short-hairpin RNA has also been reported in SKOV-3 cells, which display intrinsic cisplatin resistance^[46]^.

Molecular inhibition of USP14 by siRNAs also impaired the migratory and invasive abilities of drug-resistant cells. Of note, this effect was associated with downregulation of the receptor tyrosine kinase Axl, previously shown to be overexpressed in IGROV-/Pt 1 cells. Shen *et al.*^[47]^ suggested that the mechanism linking USP14 to cisplatin resistance involves BCL6, a transcription factor whose proteasomal degradation is prevented by the interaction with USP14. However, in IGROV-1/Pt1 cells, USP14 silencing did not decrease BCL6 levels (data not shown), suggesting that alternative mechanisms may mediate drug resistance in this context. Interestingly, in gastric cancer, cisplatin resistance is promoted by USP14 through activation of the PI3K-Akt and RAF/MEK/ERK pathways, as USP14 knockdown triggered proteasomal degradation of phospho-Akt and phospho-ERK1/2. In IGROV-1/Pt1 cells, however, we did not observe a decrease in phospho-ERK levels following USP14 silencing.

Antibody array analysis of apoptosis-related proteins revealed that USP14 silencing modulated antiapoptotic proteins in a manner consistent with enhanced cisplatin susceptibility. Specifically, Bcl-x_L_ and survivin levels were reduced in USP14-silenced cells compared with negative control siRNA-transfected cells following treatment.

Given the potential of USP14 as a therapeutic target in ovarian carcinoma, we also performed a screening to identify novel small-molecule inhibitors. To the best of our knowledge, no USP14i are currently under clinical evaluation, and the disappointing outcomes of previous clinical trials^[23]^ underscore the need for novel compounds. The first class of selective USP14i was developed by Finley’s group in 2010, exemplified by IU1^[44]^. Subsequent structural-guided optimization yielded more potent (IU1-47) and more soluble (IU-1-248) analogs^[30,48]^. More recently, Adelakun *et al.* described structurally related compounds, CID 43013232 and CID 112370349, as promising hits with stronger binding affinity to USP14 than IU1^[49]^. Here, we conducted medium-throughput screening of a compound subset from the IIT library, which contains > 13,000 small molecules. The subset was assembled through hierarchical agglomerative cluster analysis of the entire collection, followed by selection of centroid compounds from each cluster and visual inspection to exclude overly complex or redundant structures. This strategy yielded a structurally diverse panel of compounds representing the chemical space of the IIT library. Screening of this panel, which included a few analogs of known USP14i, identified ARN12502 as a USP14 inhibitor with double-digit micromolar potency. ARN12502 displayed cytotoxicity only at high micromolar concentrations in parental IGROV-1 cells (data not shown). Although its inhibitory activity is modest, ARN12502 may serve as a prototype for a new chemical class of USP14i.

In conclusion, our findings corroborate the evidence that USP14 is a valuable therapeutic target in ovarian carcinoma, particularly in cisplatin-resistant disease. Through medium-throughput screening, we identified a promising novel compound, ARN12502, that expands the repertoire of USP14i. Furthermore, molecular dynamics simulations provided insight into its binding mode, offering a basis for further medicinal chemistry efforts to design new analogs of ARN12502 with improved biological activity.

## DECLARATIONS

### Availability of data and materials

Data and materials are available upon reasonable request to the corresponding author.

### Copyright

© The Author(s) 2025.
