# Supplementary material for: Molecular targeting of the deubiquitinase USP14 to circumvent cisplatin resistance in ovarian carcinoma and identification of novel inhibitors
Source: Cancer Drug Resist. 2025 Sep 18;8:50. doi: 10.20517/cdr.2025.88 (PMC12539213; doi:10.20517/cdr.2025.88)

Supplementary Materials

Molecular targeting of the deubiquitinase USP14 to circumvent cisplatin resistance in ovarian carcinoma and identification of novel inhibitors

Cristina Corno[1], Debora Russo[2,a], Francesco Pignotti[3], Francesca De Giorgi[2], Ilaria Penna[4], Francesco Saccoliti[2,b], Matteo Costantino[1], Luca Mirra[1], Pietro Pettinari[1], Nives Carenini[1], Elisabetta Corna[1], Nunzio Perta[3], Chiara M Ciniselli[5], Pietro Pratesi[5], Rita Scarpelli[4], Fabio Bertozzi[2,c], Paolo Verderio[5], Giovanni L. Beretta[1], Giovanni Di Muccio[3], Daniele Di Marino[3], Tiziano Bandiera[2], Paola Perego[1]

[1]Unit of Molecular Pharmacology, Department of Experimental Oncology, Fondazione IRCCS Istituto Nazionale dei Tumori, Milan 20133, Italy.

[2]D3-PharmaChemistry, Istituto Italiano di Tecnologia, Genoa 16163, Italy.

[3]Department of Life and Environmental Sciences, Polytechnic University of Marche, Ancona 60131, Italy.

[4]Medicinal Chemistry and Technologies for Drug Discovery and Delivery Facility, Istituto Italiano di Tecnologia, Genoa 16163, Italy.

[5]Unit of Bioinformatics and Biostatistics, Department of Epidemiology and Data Science, Fondazione IRCCS Istituto Nazionale dei Tumori, Milan 20133, Italy.

Present address:

[a]Structural Biophysics Facility, Istituto Italiano di Tecnologia, Genova 16163, Italy.

[b]Dipartimento di Scienze della Vita, della Salute e delle Professioni Sanitarie, Università degli studi Link Campus University, Roma 00165, Italy.

[c]Computational and Chemical Biology, Istituto Italiano di Tecnologia, Genova 16163, Italy.

Correspondence to: Giovanni L. Beretta, Unit of Molecular Pharmacology, Department of Experimental Oncology, Fondazione IRCCS Istituto Nazionale dei Tumori, Milan 20133, Italy. E-mail: giovanni.beretta@istitutotumori.mi.it

Supplementary Material and Methods.

Quantitative Real-Time PCR (qRT-PCR)

Reactions were carried out in 10 µL volume with 5 µL master mix (TaqMan Universal Fast PCR Master mix, Thermo Fisher Scientific), 0.5 µL of the specific TaqMan Assay. [USP14 (Hs00193036_m1), Axl (Hs01064444_m1, GAPDH (Hs99999905_m1), RPS13 (Hs01011488_g1) Thermo Fisher Scientific]. GAPDH and RSP13 were used as housekeeping control genes. Untransfected cells were used as calibrators. A 7900HT Fast Real-Time PCR System, SDS 2.4 software and RQ manager software (Thermo Fisher Scientific) were used for reactions and data analysis through relative quantification (RQ) method, based on comparative Ct (ΔΔCt). In brief, after identification of cycle threshold (the PCR cycle at which each probe fluorescence exceeds the detection threshold, Ct), ΔCt, ΔΔCt and RQ were calculated as follows: ΔCt = Ct gene - Ct housekeeping; ΔΔCt = ΔCt sample - ΔCt calibrator and $RQ = 2^{-\Delta\Delta Ct}$.

Western blot analysis

Cells were lysed in 0.125 M Tris HCl pH 6.8 (Sigma-Aldrich), 5% sodium dodecyl sulfate (SDS, Lonza) in presence of protease/phosphatase inhibitors (25 mM sodium fluoride, 10 µg/mL pepstatin A, 1 mM phenylmethylsulfonyl fluoride, 10 µg/mL trypsin inhibitor, 12.5 µg/mL leupeptin, 30 µg/mL aprotinin, 1 mM sodium orthovanadate and 1 mM sodium molybdate, all from Sigma-Aldrich). Samples were boiled for 5 min, sonicated for 25 s and quantified with the BCA method (Pierce, Thermo Fisher Scientific). Protein extracts were fractionated onto SDS-PAGE and blotted on nitrocellulose membranes. Upon blocking in PBS with 5% (w/v) dried non-fat milk, blots were incubated with primary antibodies overnight at 4°C. Bands were revealed by enhanced chemiluminescence detection system ECL (GE Healthcare, Buckinghamshire, UK). The following antibodies were used: anti-actin and anti-β tubulin from Abcam (Cambridge, UK), anti-vinculin from Sigma-Aldrich, anti-USP14 from Bethyl Laboratories (Montgomery, Texas, USA). Secondary antibodies were from GE Healthcare. Band intensities were quantified using the Image J 1.47v software (NIH, Bethesda, MD).

Human proteome array

The analysis of the expression levels of stress-related factors was performed by the Human Apoptosis Array kit (R&D Systems, Abingdon, UK), according to the manufacturer's protocol. Briefly, 24 h after seeding, cells were exposed to 10 nM siRNA directed to USP14 (Thermo Fisher Scientific) or control siRNA (Silencer Select Negative Control #2 siRNA, Thermo Fisher Scientific), and after 48 h from the transfection start treated for 1 h with cisplatin at a concentration corresponding to the IC_{80}. Cells were harvested 24 h later and lysed for protein extraction.

Arrays were incubated overnight with protein extracts (300 µg). Following washing, arrays were incubated with a cocktail of biotinylated detection antibodies. Upon streptavidin-HRP exposure, the arrays were developed with chemiluminescent detection reagents. The levels of bound proteins correspond to the spots detected on the exposed array.

Supplementary Table 1. Characteristics of the study sample

	Frequency (N)	Percent (%)
**Diagnosis**		
Endometroid carcinoma	25	18.66
High grade serous carcinoma	80	59.7
Others[a]	29	21.64
**Stage**		
I	26	19.4
II	21	15.67
III	71	52.99
IV	16	11.94
**Grade**		
1 well differentiated	15	11.19
2 moderately differentiated	13	9.7
3 poorly differentiated	106	79.1

[a] mucinous adenocarcinoma, clear cells carcinoma, low grade serous carcinoma.

Supplementary Figure 1. USP14 relative expression distribution. Boxplot representing the expression of the USP14 according to tumor grade (A), diagnosis (B) and stage (C) on a dichotomic scale. Each box indicates the 25th and 75th centiles of the distribution. The horizontal line inside the box indicates the median and the whiskers indicate the extreme measured values.

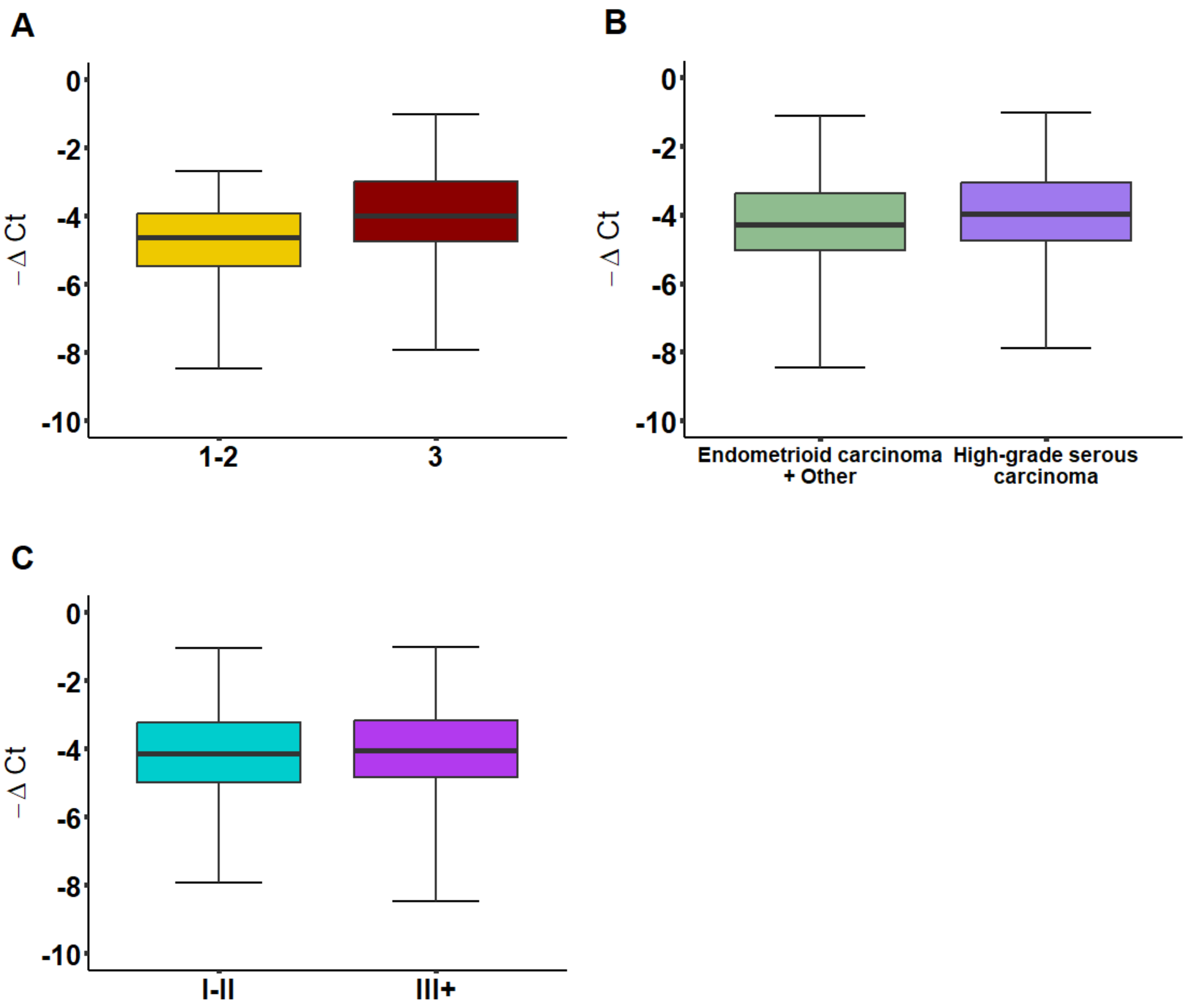

Supplementary Figure 2. USP14 mRNA expression in different ovarian carcinoma cell lines and respective resistant variants.

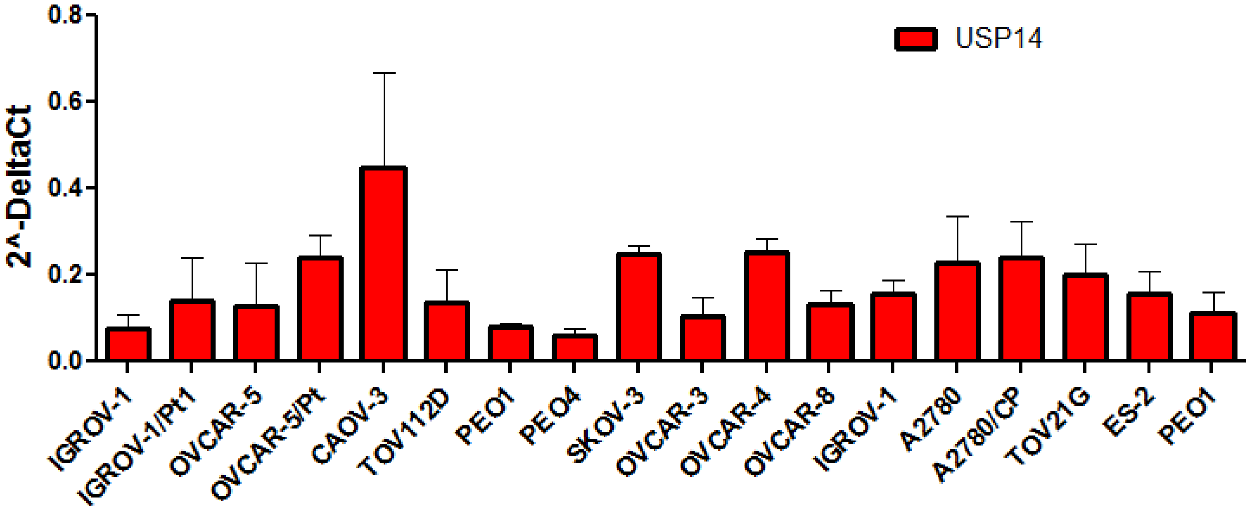

RPS13 was used as housekeeping gene.

Supplementary Figure 3. Quantification of western blot analysis reported in Figure 2. USP14 protein expression in IGROV-1 and IGROV-1/Pt1 cells after infection with USP14 or lenti ctr vector (figure 2), related to IGROV-1 parental cells (set to 1) is reported in the histogram below. Band intensity is quantified using ImageJ and normalized to actin.

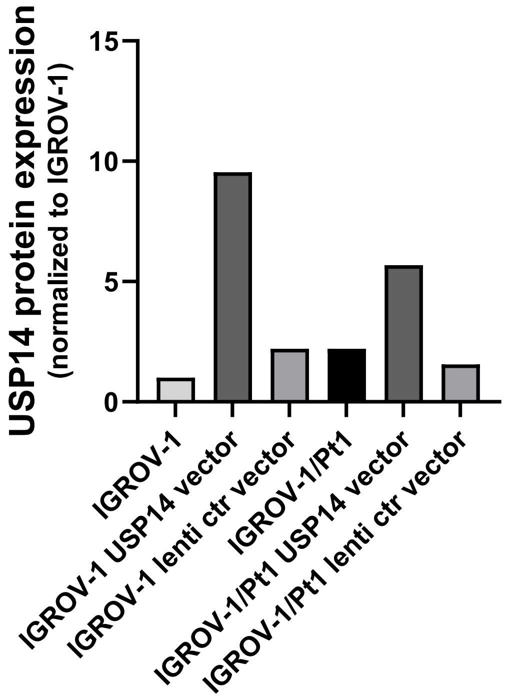

Supplementary Figure 4. Apoptosis-related proteins modulation by antibody arrays. Twenty-four h after seeding, cells were exposed to 10 nM siRNA directed to USP14 or negative control, and after 48h from the transfection start treated for 1h with ciplatin at a concentration corresponding to the IC_{80}. Cells were then harvested and lysed for protein extraction and hybridization according to the manufacturer's protocol.

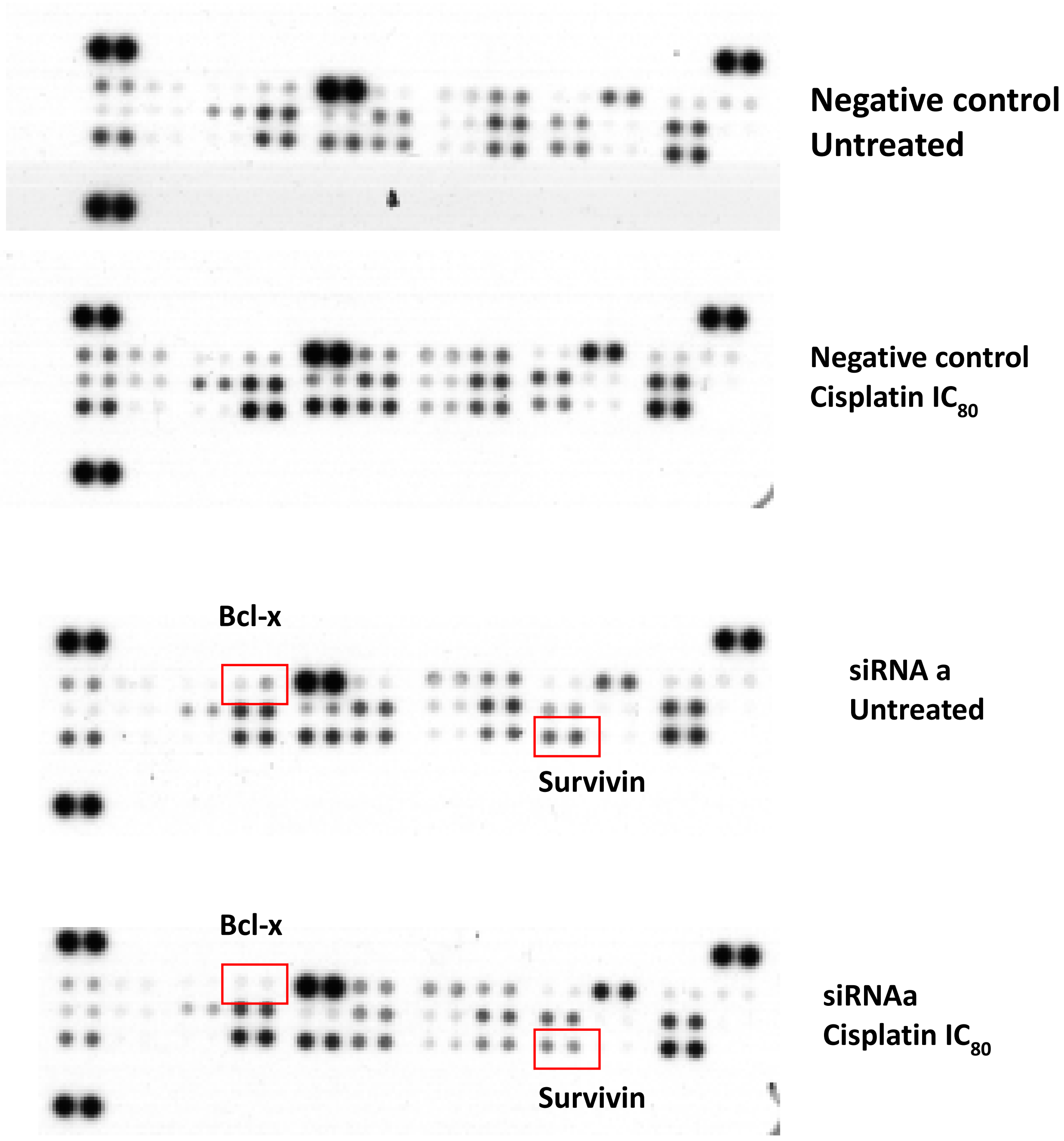

Supplementary Figure 5. a) IU1 redocking scores of Glide and DiffDock with respect to Usp14 (PDB: 6IIK). b) Images of the top 10 poses obtained from Glide and DiffDock blind docking to identify the binding pocket of CPD1, which was found to be the same of the IU family.

a)

REDOCKING GLIDE (BLIND)

Rank	State Penalty	Docking Score	Glide score	Glide emodel	RMSD (nm)
1	0.1391	-5.713	-5.852	-55.137	0.141
2	0.1391	-5.232	-5.371	-49.289	0.109
3	0.1391	-5.13	-5.27	-49.392	0.125
4	0.1391	-5.016	-5.155	-46.536	0.193
5	0.1391	-4.746	-4.885	-45.167	0.165

REDOCKING DIFFDOCK

Rank	Confidence Score	RMSD (nm)
1	-0.23	0.062
2	-0.23	0.123
3	-0.24	0.068
4	-0.24	0.061
5	-0.24	0.066

REDOCKING GLIDE (TARGET)

Rank	State Penalty	Docking Score	Glide score	Glide emodel	RMSD (nm)
1	0.1391	-5.992	-6.131	-54.871	0.104
2	0.1391	-5.505	-5.644	-49.737	0.159
3	0.1391	-5.374	-5.513	-48.779	0.149
4	0.1391	-5.371	-5.51	-47.68	0.134
5	0.1391	-5.139	-5.278	-45.563	0.069

b)

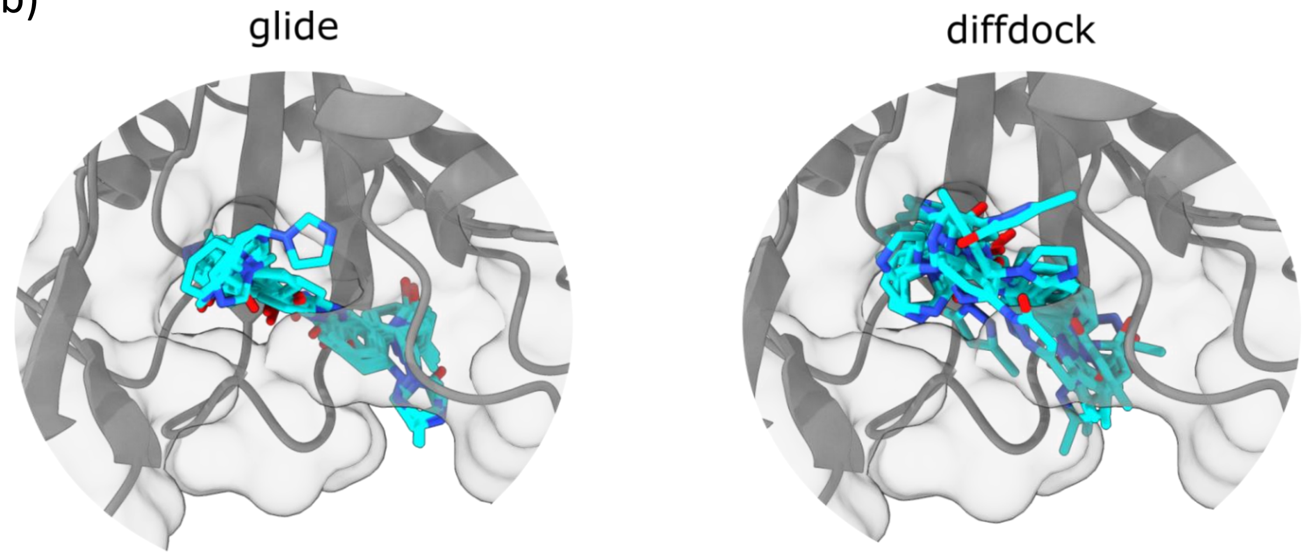

Supplementary Figure 6. Image of the USP domain of Usp14 (PDB: 7w3a) in the open blocking loops (BLs) state. The finger (cyan), palm (orange), and thumb (green) subdomains are highlighted, along with BL1 (magenta) and BL2 (blue). The zoom shows the catalytic triad (cornflower blue).

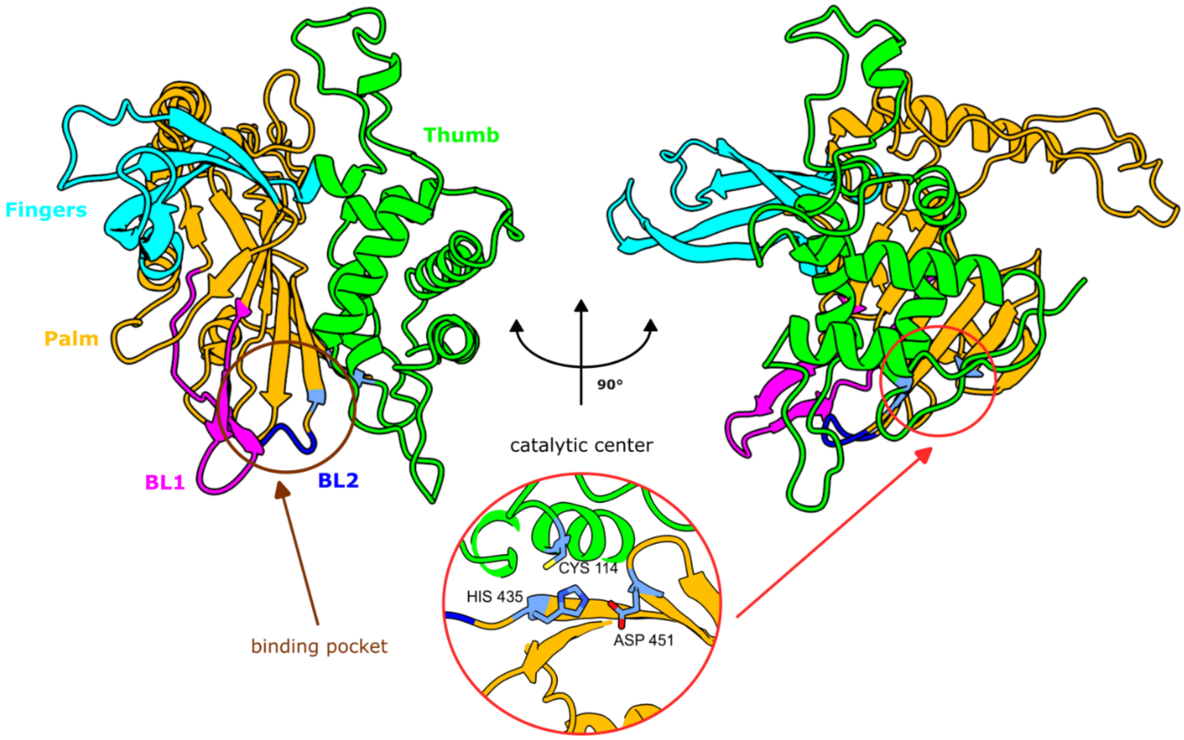

Supplementary Figure 7. a) Bar plot showing the mean emodel score of the first 29 clusters and the number of poses contained in each of them using a color scale. The poses with the most negative emodel derived from cluster 1 (cl1) and cluster 2 (cl2) were simulated. b) Distance of the compound's center of mass (COM) with respect to the binding pocket COM. It can be noted that only pose 2 (pose derived from cl2) remained stable for all the simulation time. For this latter, two additional replicas were performed, which were then analyzed in more detail in the main text.

a)

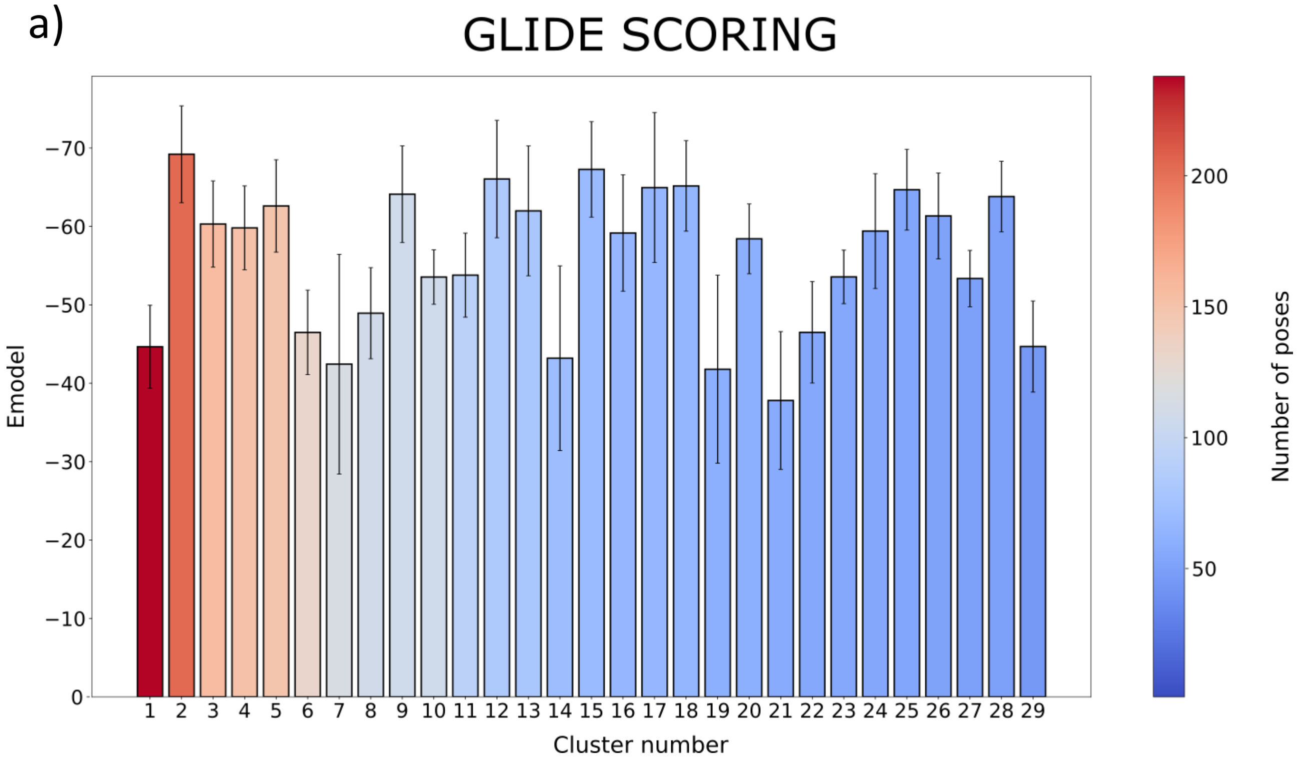

b)

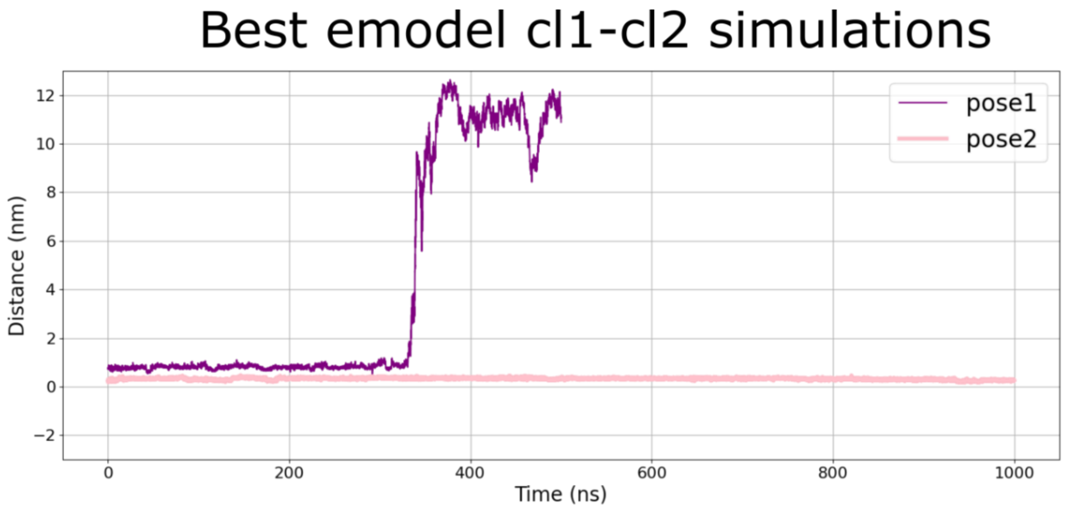

Supplementary Figure 8. Extended results calculated for the three replicas of the stable system shown in Fig. 6 of the main text and Supp. Fig. F3b. a-b) RMSD of the ligand and its distance from the center of mass of the binding pocket, relative to its configuration at 50 ns. RMSD is calculated aligning the protein backbone. A Gaussian filter is applied to smooth the curves. c) Fingerprinting analysis for each replica system, showing the type of contact of the most contacted (by the ligand) residues of the protein, along the simulation time.

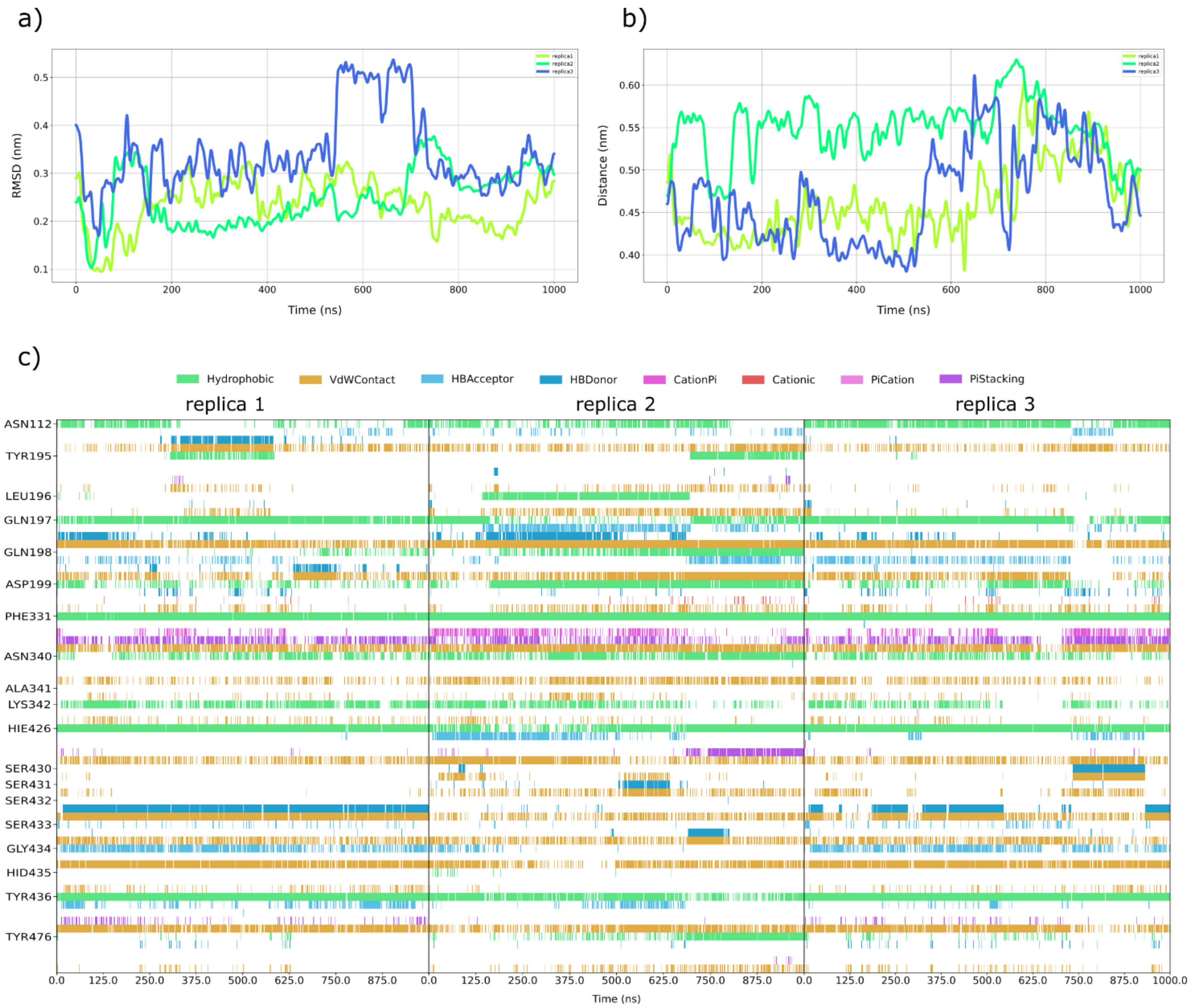

Supplement: Supplementary file 1 [file cdr-8-50-SupplementaryMaterials.pdf]
